# Calculating diabetic foot ulceration healing times using the Texas wound classification system

**DOI:** 10.1186/1757-1146-7-S2-A3

**Published:** 2014-11-14

**Authors:** Jacqueline Forss, David Till

**Affiliations:** 1 East Sussex Healthcare Trust, East Sussex, UK

## Background

Healing rates of foot ulcers are unknown with the exception of specialist centres where it is between 80-90%. The aim of this study was to ascertain average healing rates for people with diabetic foot ulceration attending a Multidisciplinary Diabetic Foot Team (MDFT) clinic by implementing the University of Texas wound classification system into clinical practice.

## Methods

All patients presenting to the MDFT with active ulceration during 2010 had their ulcer classified using the Texas system and were entered into the audit. These ulcers were then monitored until healing or until the end of the data collection period (December 2012). A patient with multiple ulcer sites had each ulcer monitored separately. The average healing rates could then be calculated and compared.

## Results

90 patients with ulcers were included with a total of 146 ulcers monitored. 32 ulcers were excluded as patients had either moved away, died, refused treatment, or withdrawn from the study due to non-concordance with treatment. 109 of the remaining 114 ulcers had healed by the end of the audit period (95.6%)

The average healing rates were:

• Overall = 16.08 weeks (range 1-74)

• Texas classification A = 10.45 weeks (range 2-20)

• Texas classification B = 12.12 weeks (range 3-30)

• Texas classification C = 14.2 weeks (range 1-42)

• Texas classification D = 28.4 weeks (range 4-74)

In relation to amputation rates:

• Texas classification A=0

• Texas classification B = 1 minor

• Texas Classification C = 2 minor

• Texas classification D = 3 minor and 2 major amputations.

## Conclusion

The Texas classification system was easy to use in clinical practice. The data demonstrates that average healing time varied according to the ulcer classification at presentation. To our knowledge, this has not previously been investigated. In addition, overall healing rates are higher than previously published data suggests, whereas amputation rates are similar to those reported using this classification system. It is recommended that the audit be continued to increase the sample sizes to identify any intergroup variation in mean healing times.
